# Siah2 modulates sex-dependent metabolic and inflammatory responses in adipose tissue to a high-fat diet challenge

**DOI:** 10.1186/s13293-019-0233-y

**Published:** 2019-04-15

**Authors:** Sujoy Ghosh, Jessica L. Taylor, Tamra M. Mendoza, Thanh Dang, David H. Burk, Yongmei Yu, Gail Kilroy, Z. Elizabeth Floyd

**Affiliations:** 1 Pennington Biomedical Research Center, Baton Rouge, LA 70808 USA; 2 Cardiovascular and Metabolic Disease Program and Center for Computational Biology, Duke-NUS Graduate Medical School, Singapore, Singapore

**Keywords:** Adipose tissue, Estrogen, Estrogen receptor, Estrogen-related receptor, Inflammation, Obesity, Sex, Siah2, Ubiquitin

## Abstract

**Background:**

The obesity-related risk of developing metabolic syndrome is higher in males than in females of reproductive age, likely due to estrogen-mediated reduced adipose tissue inflammation and fibrosis with hypertrophied adipocytes. Depletion of the ubiquitin ligase Siah2 reduced white adipose tissue inflammation and improved glucose metabolism in obese male mice. Siah2 is a transcriptional target of estrogen, but data is lacking about the effect of Siah2 on adipose tissue of females. We therefore evaluated the impact of Siah2 deficiency on white and brown adipose tissue in females of reproductive age.

**Methods:**

Body composition, adipose tissue morphology, brown adipose tissue gene, and protein expression and adipocyte sizing were evaluated in wild-type and Siah2KO female and male mice fed a low-fat or high-fat diet. Glucose and insulin tolerance, fasting glucose, insulin, fatty acids and triglycerides, and gene expression of inflammation markers in perigonadal fat were evaluated in wild-type and Siah2KO female mice. Microarray analysis of brown fat gene expression was carried out in both sexes. Statistical analysis was assessed by unpaired two-tailed *t* test and repeated measures ANOVA.

**Results:**

Siah2 deficiency improves glucose and insulin tolerance in the presence of hypertrophied white adipocytes in high-fat-fed female mice with percent fat comparable to male mice. While previous studies showed Siah2KO reduces the white adipose tissue inflammatory response in male mice, the response in females is biased toward the upregulation of M2-like markers in white adipose tissue. In contrast, loss of Siah2 leads to increased whitening of brown fat in males, but not in females. This corresponded to increased expression of markers of inflammation (*F4/80*, *Ccl2*) and thermogenic genes (*Pgc1alpha*, *Dio2*, *Ucp-1*) and proteins (PGC-1α, UCP-1) in females. Contrary to expectations, increased expression of thermogenic markers in females was coupled with a downregulation of ERalpha and ERRgamma protein levels.

**Conclusions:**

The most striking sex-related effect of Siah2 deficiency is reduced whitening of brown fat in high-fat-fed females. Protection from accumulating unilocular adipocytes in the brown fat corresponds to increased expression of thermogenic genes and proteins in female, but not in male mice. These results raise the possibility that Siah2 contributes to the estrogen-related effects on brown fat function in males and females.

**Electronic supplementary material:**

The online version of this article (10.1186/s13293-019-0233-y) contains supplementary material, which is available to authorized users.

## Background

Obesity increases the risk of developing metabolic syndrome, a collection of risk factors for type 2 diabetes that includes insulin resistance and dyslipidemia. The connection between obesity and metabolic dysfunction is more common in males than females of reproductive age in rodents [1–3] and humans [4, 5]. Although women have an overall higher percent body fat, increased lower body subcutaneous adipose tissue in women is thought to confer protection against obesity-related insulin resistance that is typically associated with abdominal visceral fat more common in men [6–9]. However, this protection diminishes in postmenopausal females as visceral fat increases, pointing to the importance of sex hormones in influencing fat distribution [10, 11]. Although estrogen-driven accumulation of subcutaneous fat protects against obesity-related insulin resistance [10], recent trends indicate a rise in metabolic syndrome among younger females, mainly driven by increased intra-abdominal visceral obesity [12]. This reinforces the strong correlation between abdominal adipose tissue and the adverse metabolic consequences of obesity in males or females.

Metabolic complications with obesity arise from the inability of adipose tissue to expand and safely store the excess lipids. Adipose tissue can expand by producing new adipocytes (hyperplasia) or increasing the volume of existing fat cells (hypertrophy). Abdominal adipose tissue expands by either route, but is less able to generate new adipocytes than subcutaneous adipose tissue [13]. When the lipid storage capacity of the hypertrophied adipocytes is exceeded, the lipid is stored in the skeletal muscle and liver, leading to insulin resistance [14, 15]. This coincides with the increased release of fatty acids from the enlarged adipocytes, recruitment of macrophage to the adipose tissue, and higher expression of pro-inflammatory proteins that signifies chronic, low-grade inflammation in the adipose tissue.

Estrogens affect the relationship between adipocyte hypertrophy, adipose tissue inflammation and insulin responsiveness in males and females [9]. Estrogen-responsive receptor alpha (ERα) depletion in visceral fat leads to adipocyte hypertrophy and adipose tissue inflammation in male and female mice [16]. However, when ERα is deleted specifically in adipocytes, sex-related differences emerge in which adipose tissue expansion via adipocyte hypertrophy occurs in females, but not male mice. Even so, the females are protected from adipose tissue inflammation and impaired glucose metabolism despite adipocyte hypertrophy [16].

Obesity-related changes in adipocyte size and adipose tissue inflammation also occur in brown adipose tissue, and sex-related differences in adipose tissue extend to brown fat as well [17]. Women have increased brown adipose tissue mass compared to men [18, 19], and estrogen activates brown fat thermogenesis, but there is no clear evidence of higher energy expenditure in women. Brown adipose tissue’s ability to use lipids as fuel to drive adaptive thermogenesis is negatively impacted by obesity as the brown fat undergoes “whitening” with the accumulation of large, unilocular lipid droplets characteristic of white fat [20]. In male mice, this is accompanied by brown adipose tissue inflammation as macrophages surround the dysfunctional “whitened” brown adipocytes [21], forming the characteristic “crown-like structure” indicative of macrophages surrounding lipid droplet remnants from dead adipocytes [22]. While the anti-inflammatory properties of estrogen are well-known [23, 24], sex-related differences in estrogen signaling in brown adipose tissue with obesity are not well described.

Our earlier studies of the mammalian homolog of the *Drosophila* ubiquitin ligase seven-in-absentia-2 (Siah2) in adipose tissue from obese male mice showed that Siah2 deficiency leads to adipocyte hypertrophy in white adipose tissue, but protects against adipose tissue inflammation and the associated insulin resistance [25]. Siah2 interacts with the peroxisomal proliferator-activated receptor gamma (PPARγ) [26], a nuclear receptor that regulates lipid metabolism as well as inflammatory responses in adipose tissue [27], and selectively regulates PPARγ activity in gonadal adipose tissue [25]. Siah2 is also a transcriptional target of the nuclear receptor ERα. In ERα-positive breast cancer cells, estrogen stimulates gene expression by upregulating Siah2 transcription and stimulating Siah2-mediated N-CoR degradation [28]. Estrogen-related regulation of Siah2, and its previously observed effects on white adipose tissue, prompted us to examine sex-dependent differences in white and brown adipose tissue inflammation in diet-induced obesity in a systemic Siah2-deficiency (Siah2KO) mouse model.

Here, we show that loss of Siah2 protects against impaired glucose metabolism and disrupts the connection between hypertrophied adipocytes and adipose tissue inflammation in the white adipose tissue of the high-fat-fed females, similar to our earlier reports in male mice. Most strikingly, Siah2 deficiency upregulates expression of *Pgc1a*, *Dio2*, and *Ucp1* in female, but not in male brown fat mice. The change in thermogenic gene expression corresponds to increased protein expression of PGC1α and UCP1 and less whitening of the female brown fat than observed in the male mice. Unexpectedly, enhanced markers of brown fat thermogenesis in the HFD-fed females correspond to substantially reduced protein expression of the nuclear receptors ERα and ERRγ that promote brown fat thermogenesis [29, 30]. This suggests that sex-related modulation of Siah2 activity in brown fat may act to dampen thermogenic responses to chronic overnutrition in females by regulating ERα and ERRγ protein levels in brown fat.

## Methods

### Experimental animals

Siah2KO mice were generated and maintained as described [25, 31]. Wild-type C57BL/6J mice were obtained from Jackson Laboratories. The female mice were reproductively intact. All animal experiments were approved by the Pennington Biomedical Research Center Animal Care and Use Committee (protocol #1030). The animals were multi-housed with a 12-h light-dark cycle at 24 °C. At 4 weeks of age, wild-type and Siah2KO male and female mice of similar body weight within each sex were randomly assigned (*n* = 8–10/group) to a defined 10% low-fat (LFD; 10% kcal fat, Research Diets, #D12450H, sucrose matched to the HFD) or 45% high-fat (HFD; 45% kcal fat, Research Diets, #D12451) diet and were fed ad libitum for 4 months thereafter. Body weight was measured weekly and body composition was measured bi-weekly by NMR. At the end of the study, the mice were euthanized between 8 and 11 AM.

### Glucose and insulin tolerance tests

For the glucose (GTT) and insulin (ITT) tolerance tests, the amount of glucose or insulin administered was normalized to fat-free mass [32], which did not vary significantly among groups (20.1 −/+ 0.13 g) at 12 weeks on each diet. Mice were fasted 4 h prior to administering 2 g/kg fat-free mass of glucose/mouse (GTT) or 1 U/kg fat-free mass insulin/mouse (HumulinR) (ITT) by intraperitoneal injection.

### Blood chemistry

Fasting serum glucose levels were measured using a Breeze2 glucometer (Bayer, Leverkusen, Germany). Fasting insulin and leptin levels were assayed via ELISA (Crystal Chem). Serum nonesterified fatty acids (Abcam) and triglycerides (Eagle Diagnostics) levels were assayed according to manufacturers’ instructions.

### Microarray analysis

Brown adipose tissue RNA (RNA integrity number ≥ 8) was analyzed for gene expression on Illumina MouseRef-8v2.0 expression arrays. RNA from eight to ten animals/group was combined into three pooled samples/group. Samples from male and female animals were analyzed separately. Raw gene expression signals were background adjusted and quantile normalized using GenomeStudio (V2011.1.Illumina Inc.). For each sample, probes with detection *p* value < 0.05 were considered “expressed.” These probes were log transformed (base 2), and treatment-specific fold changes were computed as log ratios. The statistical significance of differential expression was ascertained by a regularized *t* test, based on Bayesian probability models [33]. All statistical analyses were controlled for multiple testing via the false discovery rate (FDR) [34]. The microarray dataset was submitted to Gene Expression Omnibus (GEO) data repository (GSE123990).

### Over-representation analysis

Over-representation analysis (ORA) of biological functions and putative upstream regulators was carried out by subjecting a pre-filtered list of 333 BAT differentially expressed genes for females and 415 genes for males (absolute fold change > 1.3 and nominal *p* value < 0.05) to Ingenuity Pathway Analysis tool (IPA, QIAGEN Redwood City). Reference gene sets corresponding to “biological functions” (as defined in the Ingenuity Knowledge Base) were analyzed for statistically significant over-representation. Additionally, predictions of changes in the activity status of upstream transcription factors that would be consistent with the observed gene expression changes were also carried out. Biological functions and upstream regulators with a *z* score > 2.0 or <− 2.0 were considered to be activated or inhibited, respectively (http://pages.ingenuity.com/rs/ingenuity/images/0812%20downstream_effects_analysis_whitepaper.pdf). Statistical significance of over-represented gene sets was ascertained via Fisher’s exact test and corrected for multiple testing via the Benjamini-Hochberg procedure [34].

### Quantitative PCR

Total RNA was purified from inguinal, gonadal, and brown adipose tissue, (200 ng) reverse transcribed, and real-time PCR performed with TaqMan chemistry as described [25]. The results were normalized to hypoxanthine-guanine phosphoribosyltransferase (HPRT), where Δ*C*_*T*_ ≤ −/+ 0.5 within each sex [35] for the males and females separately due to significant sex-related differences in housekeeping gene expression and analyzed using the 2^−ΔΔCT^ method with wild-type values used as the calibrator. The gene list is provided in Additional file 1.

### Preparation of whole cell extracts and immunoblotting

Adipose tissue was homogenized in a denaturing buffer and processed for immunoblotting as described [25]. Nitrocellulose membranes were incubated with antibodies (Additional file 2) for 1–2 h at room temperature or overnight at 4 °C. MemCode staining of the nitrocellulose and β-actin levels were used to confirm equal protein content in each lane.

### Immunohistochemistry and immunostaining

Adipose tissue was fixed in 10% formalin, then embedded in paraffin, sectioned onto slides, and stained in hematoxylin and eosin (H&E). Adipose tissue collagen content and fibrosis was determined by trichrome staining. H&E-stained inguinal and epididymal adipose tissue and laminin stained brown adipocytes (see Additional file 3 B) were analyzed using Image J software programmed to measure the area of each adipocyte based on size and shape exclusion limits. Adipocytes or adipocyte remnants surrounded by crown-like structures were manually excluded from analysis. The number of adipocytes counted/experimental condition ranged from 413 to 8442. The number of adipocytes/fat pad was approximated by converting adipocyte area to a spherical volume, assuming a circular structure for each adipocyte, and then calculating the number of adipocytes/cm^3^. After converting the fat pad weight to a volume by assuming the density of each fat pad as equivalent to lipid content at 0.915 g/cm^3^, the total volume of each fat pad number was divided by the adipocytes/cm^3^ to determine the number of adipocytes/fat pad. A small error is introduced in the calculation as this method does not account for the difference in percent lipid content in gonadal versus inguinal fat depots.

### Statistical analysis

Normal distribution of glucose and insulin levels, food intake, and body weight was assessed using the D’Agostino-Pearson omnibus normality test. Statistical significance for body weight, GTT, and ITT was determined using repeated measures ANOVA. Statistical significance for all other data was determined using an unpaired two-tailed t test. JMP Pro 10.0 (SAS Institute) and GraphPad Prism 5 softwares were used for statistical analyses. Variability was expressed as the mean −/+ standard deviation.

## Results

**Fig. 1:**
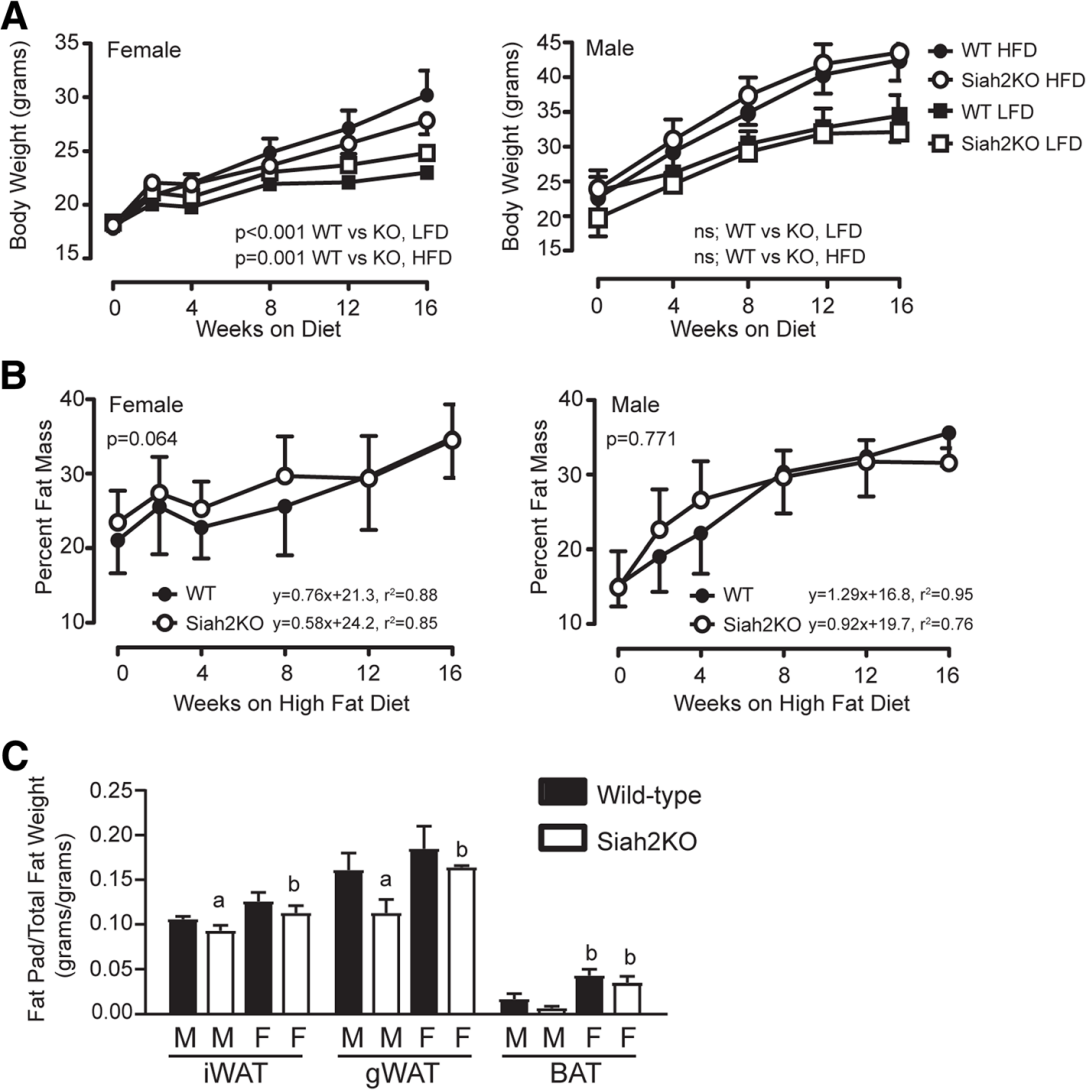
Wild-type and Siah2KO female mice fed a high-fat diet have percent fat mass comparable to male mice, but higher levels of brown fat relative to total fat mass. **a** Body weight, **b** percent fat mass, and **c** fat pad weight/total fat mass were measured in the wild-type (WT) and Siah2KO female and male mice fed a defined low (LFD)- or high (HFD)-fat diet over 16 weeks. Statistical significance was determined using repeated measures ANOVA in **a** and **b** and two-tailed, unpaired *t* test in **c**, a, *p* < 0.05; within sex comparison between genotypes. b, *p* < 0.05; between sex comparison of related genotype

We previously found that despite impaired adipogenesis [36], Siah2KO male mice become obese when challenged with a high-fat diet (HFD) [25]. To further determine if the Siah2KO phenotype is sex-related, we compared body weight and percent fat mass in female and male wild-type and Siah2KO mice fed a low-fat diet (LFD) or HFD for 4 months (Fig. 1a). In contrast to the male Siah2KO mice, body weight gain was attenuated in the female Siah2KO mice compared to the wild-type female mice on the HFD, but not the LFD (Fig. 1a). Female mice of both genotypes had higher adiposity at baseline (Fig. 1b), but the higher rate of fat mass deposition in the male mice resulted in comparable percent fat mass within 1 month of initiating the high-fat diet. However, loss of Siah2 in the males was associated with lower percent fat mass at 4 months. This is reflected in lower fat mass for white and brown adipose tissue relative to total fat mass (Fig. 1c). This did not occur in the HFD-fed Siah2KO female mice, resulting in a significant difference in relative fat mass of gonadal and brown adipose tissue between the male and female Siah2KO mice. Interestingly, the amount of brown fat mass relative to total fat mass in the females was substantially higher than observed in males independent of genotype.

**Fig. 2:**
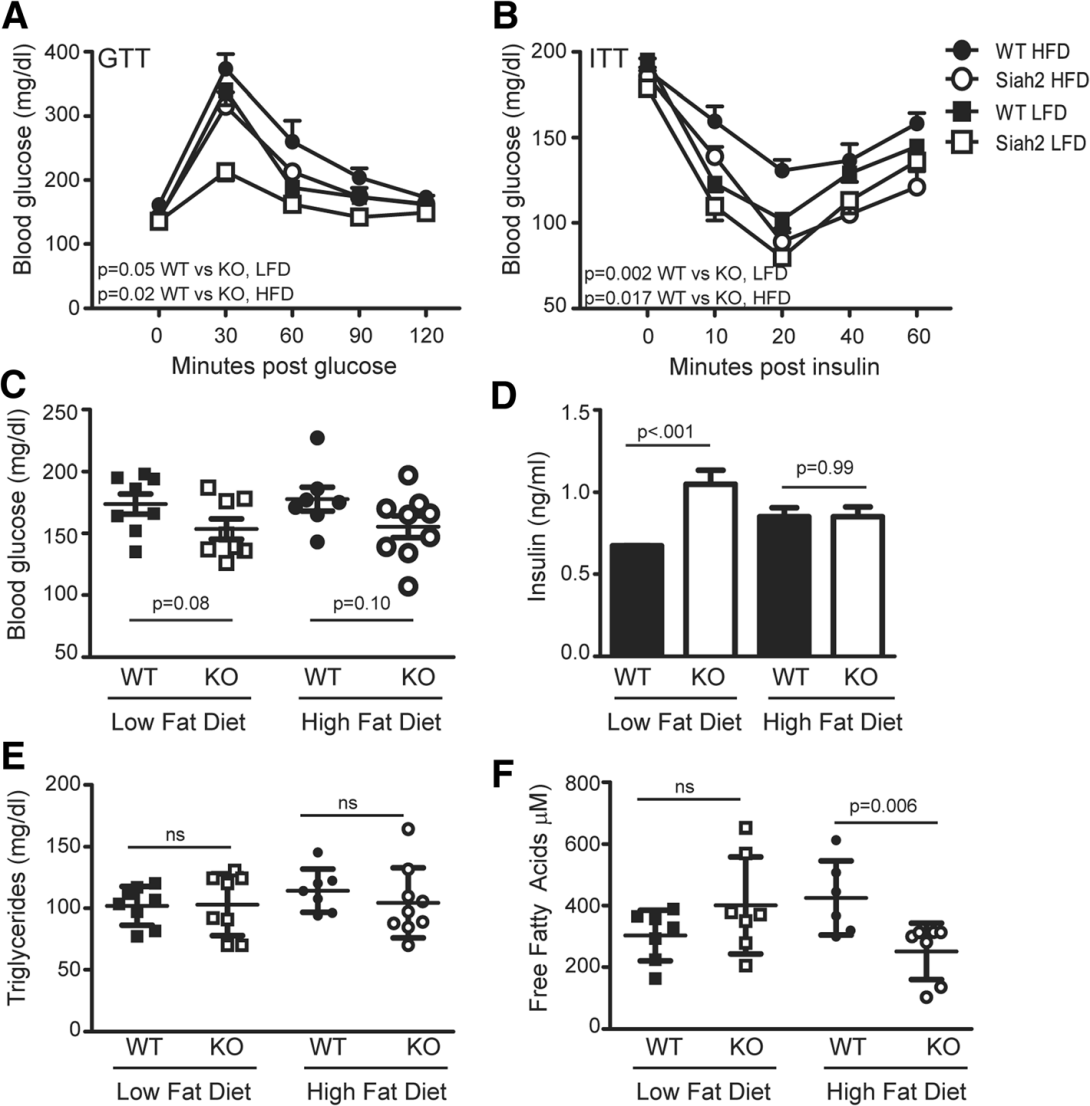
Carbohydrate metabolism is improved in the lean or obese Siah2KO female mice. **a** Glucose tolerance and **b** insulin tolerance testing were carried out at 12 weeks on the LFD or HFD. **c** Fasting blood glucose, **d** insulin, **e** triglycerides, and **f** free fatty acid levels were assayed after 16 weeks on the LFD or HFD in the female mice. Statistical significance was determined using repeated measures ANOVA in **a** and **b** and two-tailed, unpaired *t* test in **c**–**f**

Although the female wild-type and Siah2KO mice had higher adiposity than male mice at baseline and gained 50–60% of their initial body weight on the HFD, they remained glucose (Fig. 2a) and insulin tolerant (Fig. 2b). The increased responsiveness to insulin in Siah2KO females on the low- or high-fat diet was comparable to our previous findings with Siah2KO males [25] and (Additional file 4 A–C). Unlike our earlier results in male mice, Siah2 deficiency did not correspond to lower fasting glucose or insulin levels in the female mice (Fig. 2c, d). However, loss of Siah2 resulted in a twofold increase in insulin levels with the low-fat diet (Fig. 2d). Like the males in our earlier study, triglyceride levels were unchanged by diet or genotype (Fig. 2e), but increased fat mass in the Siah2KO females on high fat correlated with significantly reduced circulating free fatty acids (Fig. 2f), suggesting an improved ability of adipose tissue to store lipids.

**Fig. 3:**
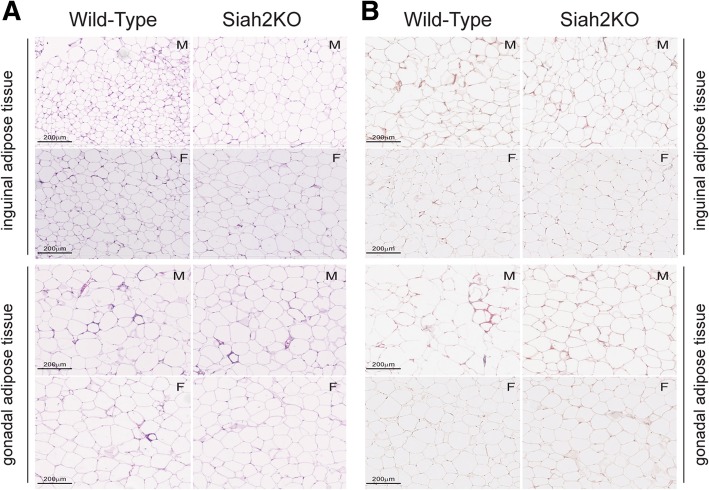
Female Siah2KO adipocytes increase in size with HFD in white adipose tissue, but accumulate fewer crown-like structures and less fibrosis compared to male white adipose tissue. **a** H&E and **b** trichrome staining of HFD male (M) and female (F) wild-type and Siah2KO inguinal and perigonadal fat

**Fig. 4:**
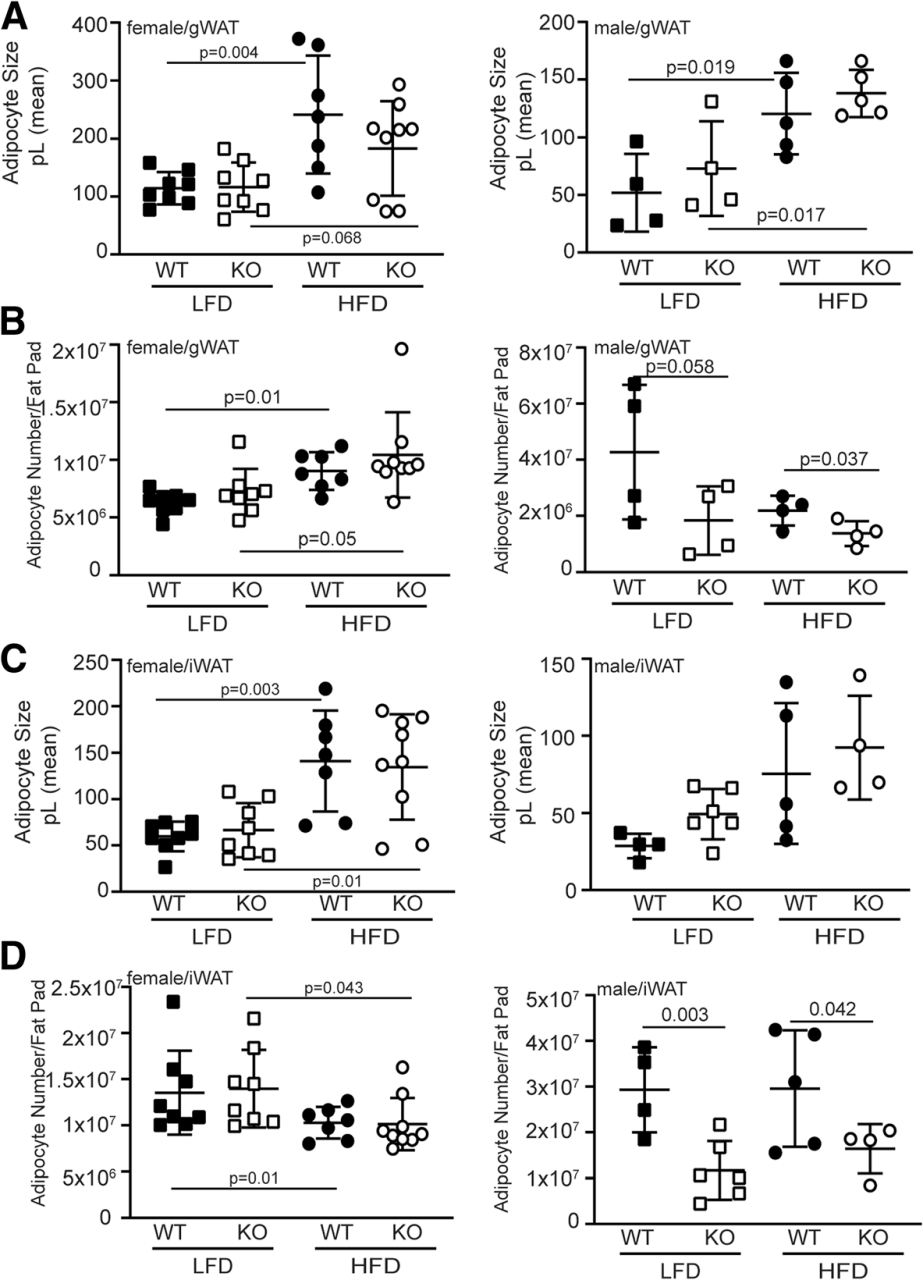
Siah2KO affect adipocyte size and number in male, but not female gonadal and inguinal fat. Adipocyte size (area) in the LFD and HFD-fed female and male wild-type (WT) or Siah2KO (KO) mice was determined by automated cell counting of H&E stained tissue using Image J software. Adipocyte number/fat pad was estimated by converting the adipocyte area to an adipocyte volume (pL) and converting fat pad weight to a volume using the density of lipids. **a** Adipocyte volume (pL) and **b** adipocyte number/fat pad for gonadal fat. **c** Adipocyte volume (pL) and **d** adipocyte number/fat pad for inguinal fat. Statistical significance was determined using two-tailed, unpaired *t* test

Healthy adipose tissue expansion with excess calorie intake requires expansion via enlarging existing adipocytes and production of new, small adipocytes that increase the capacity to store neutral lipids as adipocytes [37]. Adipose tissue expansion via hypertrophy of existing adipocytes is associated with fibrosis and recruitment of pro-inflammatory immune cells to the adipose tissue, leading to insulin resistance as the adipocytes release free fatty acids into circulation [38]. In our earlier study, a striking feature of the adipose tissue in the Siah2KO male mice was fewer crown-like structures and less adipose tissue fibrosis although the adipocytes were larger in the HFD-fed Siah2KO males compared to HFD-fed wild-type males [25]. These morphological changes were coupled with reduced expression of a wide range of genes that regulate inflammation in adipose tissue [25] (Additional file 4 D). In this study, we found that Siah2 deficiency in the HFD-fed females was also associated with fewer crown-like structures in the gonadal fat pad (Fig. 3a). The female gonadal fat (gWAT) had fewer crown-like structures independent of genotype when compared to male mice, but crown-like structures in the gWAT were further decreased in the Siah2KO females. This corresponded to less fibrosis in the female gonadal fat regardless of genotype (Fig. 3b) and larger adipocytes in the gWAT and iWAT of the females, as seen in the males (Fig. 4 a, c). Thus, depletion of Siah2 in the females resulted in a substantial decrease in morphological indicators of inflammation in white adipose tissue depots even though adipocyte size was increased with the high-fat diet. Our approximation of the number of adipocytes/fat pad indicated increased numbers as well as increased adipocyte size in the female gonadal fat, whereas in males, adipocyte hypertrophy was not accompanied by increased numbers (Fig. 4 b). The increased adipocyte size in the inguinal fat of both sexes corresponded to the reduced numbers of adipocytes in each fat pad (Fig. 4d), suggesting the subcutaneous fat tissue expanded via enlarging existing adipocytes in both sexes.

**Fig. 5:**
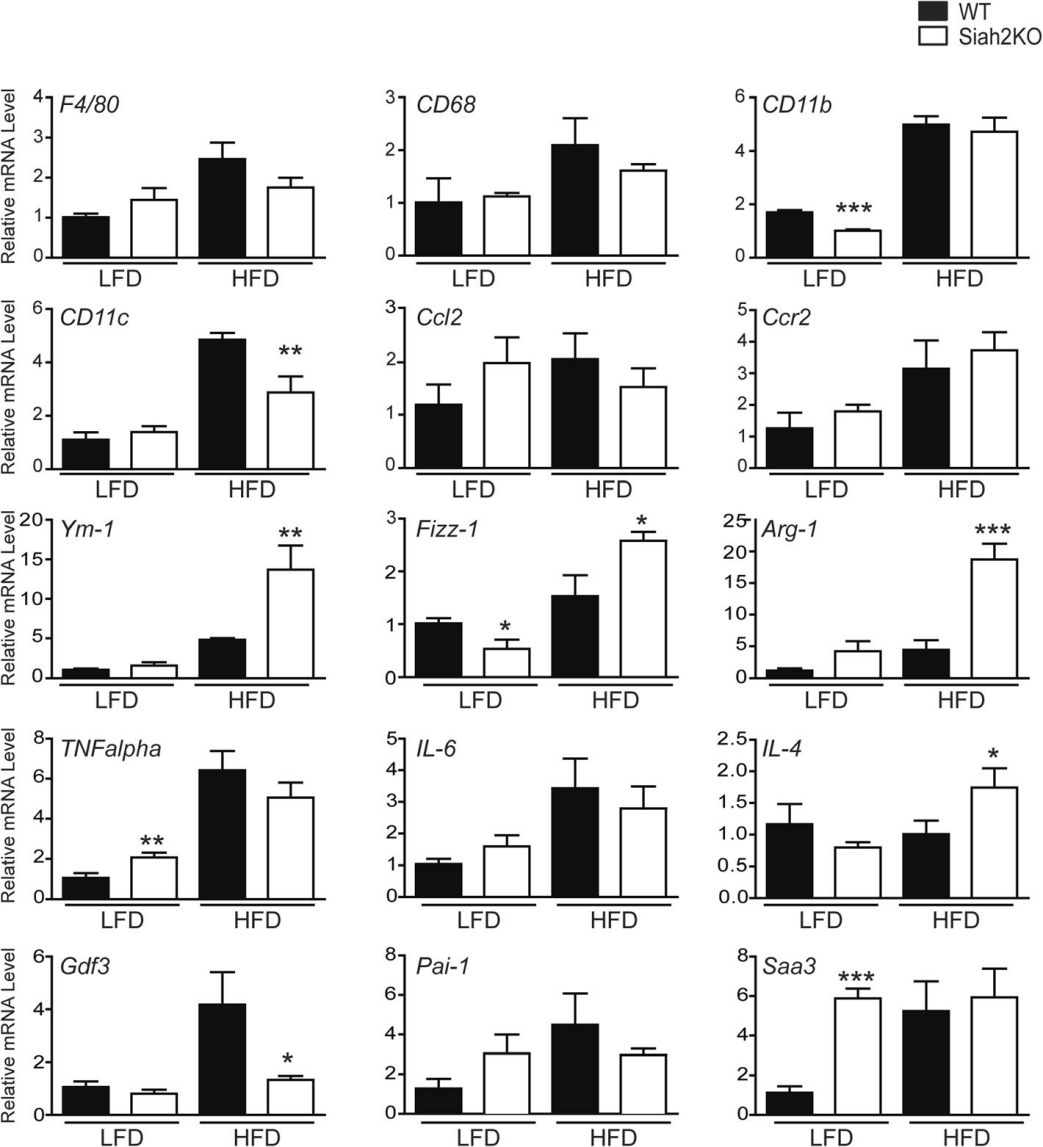
Siah2 regulates mRNA expression of markers of gonadal adipose tissue inflammation in female mice. Gene expression of markers of inflammation, cytokines, and chemokines was assayed in the perigonadal adipose tissue of wild-type (WT) and Siah2KO (KO) female mice after 16 weeks on the LFD or HFD using real-time qRT-PCR. Statistical significance of Siah2KO compared to wild-type within diet, * *p* < 0.05, *** *p* < 0.001

We anticipated that the absence of signs of adipose tissue dysfunction related to inflammation would be reflected in reduced gene expression of inflammation markers in the females. However, as shown in Fig. 5, this was not the case in the gonadal fat of the female Siah2KO mice. Unlike our previous results in male Siah2-deficient mice [25], there was no decrease in macrophage recruitment in the female visceral fat with the HFD (*F4/80*, *Cd68*, *Cd11b*). Although induction of a proinflammatory marker of M1-like macrophage (*CD11c*) was attenuated in the HFD-fed Siah2KO females, the expression of pro-inflammatory mediators was either unchanged (*Ccl2*, *Ccr2*, *Tnfalpha*, *IL-6*) or increased (Pai-1, Saa3) in contrast to that of male HFD-fed Siah2KO mice **(**Additional file 4 D). Notably, Siah2 deletion increased *Pai-1* and *Saa3* gene expression in the insulin-sensitive females independent of diet. However, genes associated with M2-like/homeostatic macrophage (*Ym-1*, *Fizz-1*, *Arg-1*, *IL-4*) were robustly upregulated only in the HFD-fed Siah2Ko females.

**Fig. 6:**
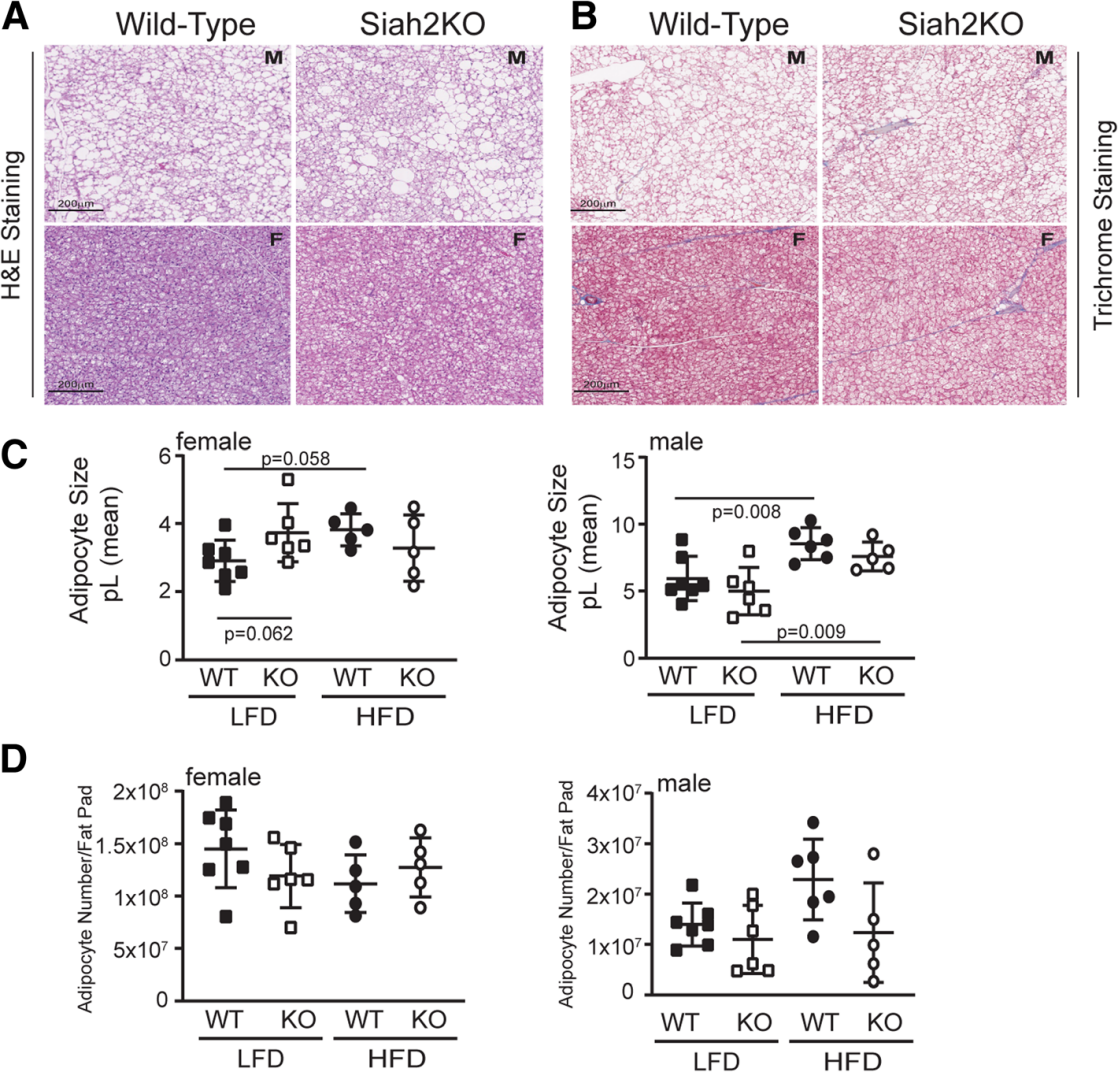
Female brown adipose tissue accumulates less unilocular fat than males on a HFD. **a** H&E and **b** trichrome staining of brown fat in wild-type and Siah2KO male (M) and female (F) mice fed a HFD for 16 weeks. **c** Adipocyte volume (pL) in the LFD and HFD-fed female wild-type (WT) or Siah2KO (KO) mice was based on adipocyte area determined by automated cell counting using Image J software of laminin stained brown adipose tissue. **d** Adipocyte number/fat pad was estimated by converting adipocyte area to an adipocyte volume (pL) and fat pad weight to a volume using the density of lipids. Statistical significance was determined using two-tailed, unpaired *t* test

While much of the focus on obesity-induced adipose tissue inflammation has centered on visceral fat depots, inflammatory responses also occur in brown adipose tissue in response to obesity [21, 39, 40]. In this study, we noted that brown fat mass relative to total fat mass in the HFD-fed wild-type or Siah2KO females was significantly higher than the males (Fig. 1c). H&E and trichrome staining of the brown adipose tissue of the wild-type and Siah2KO males and females showed striking differences in their responses to HFD. As shown in Fig. 6a, brown fat from HFD-fed wild-type and Siah2KO males accumulated unilocular adipocytes with obesity, and brown fat whitening was increased further with Siah2 deficiency. Unilocular adipocyte accumulation was substantially lower in the HFD-fed wild-type and Siah2KO females, with no additional increases in the knockout animals (an enlarged view is shown in Additional file 3 A). Trichrome staining for fibrosis in the brown fat indicated minimal fibrotic changes in the brown fat in either genotype or sex (Fig. 6b). Consistent with accumulating large lipid droplets, the brown adipocytes in the HFD-fed wild-type and Siah2KO males were substantially larger than adipocytes in the HFD-fed female (Fig. 6c). We did not detect a statistically significant diet-dependent change in the number of adipocytes/brown fat pad in either sex or genotype (Fig. 6d). To look more closely at the sex-dependent differences in brown fat (BAT) from the WT and Siah2KO males and females, we carried out a microarray analysis of the brown fat obtained from the HFD-fed mice.

**Fig. 7:**
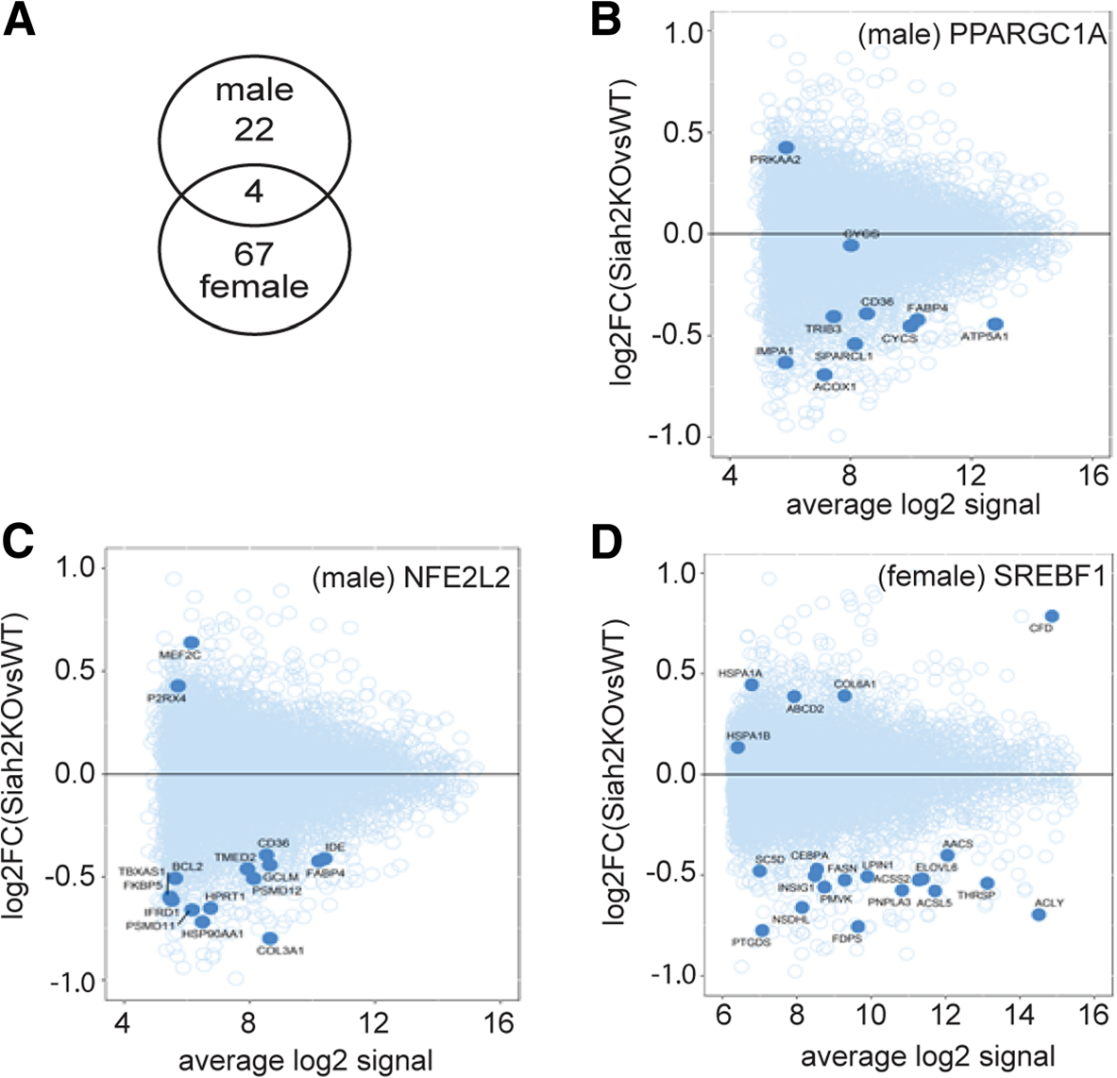
Microarray analysis of brown fat transcriptomics in HFD-fed male and female mice. **a**–**d** Analysis of gene expression in male and female BAT samples. **a** Overlap of differentially expressed genes (nominal *P* < 0.001, absolute fold-change > 1.5) in male and female samples. **b**–**d** Mean-average plots for gene targets of transcription factors *Nfe2l2* and *Ppargc1a* (male BAT samples) and *Srebf1* (female BAT samples), based on over-representation analysis in Ingenuity Pathway Analysis tool. Transcription factor target genes are shown as solid circles, whereas the remaining genes on the microarray are shown as open circles

Gene expression analysis identified a total of 26 genes that were differentially regulated in males and 71 genes in females at a nominal *p* value < 0.001 and absolute fold change > 1.5. Of these, only four genes (*Rab4a*, *Erich6*, *Entpd4*, *and LOC329575*) were differentially expressed in common between male and female BAT samples (Fig. 7), suggesting largely sex-dependent transcriptomic responses in BAT. Gene set overrepresentation analysis in IPA predicted an inhibition of transcription factors related to energy metabolism (*Ppargc1a*) (Fig. 7b) or inflammatory process (*Nfe2l2*, *Cepbp*) (Fig. 7c) in Siah2-deficient male samples, whereas Siah2-deficient female samples showed a predicted inhibition of the lipogenic transcription factors *Srebf1* and *Srebf2* (Fig. 7d). These findings are consistent with phenotypic observations, e.g., the inhibition of *Ppargc1a* mRNA signaling in male Siah2KO BAT are likely to reduce fatty acid oxidation and contribute toward whitening, and reductions in *Nfe2l2*- or *Cepbp*-encoded proteins are likely to contribute to the observed lower inflammatory tone of HFD-fed male Siah2KO samples.

**Fig. 8:**
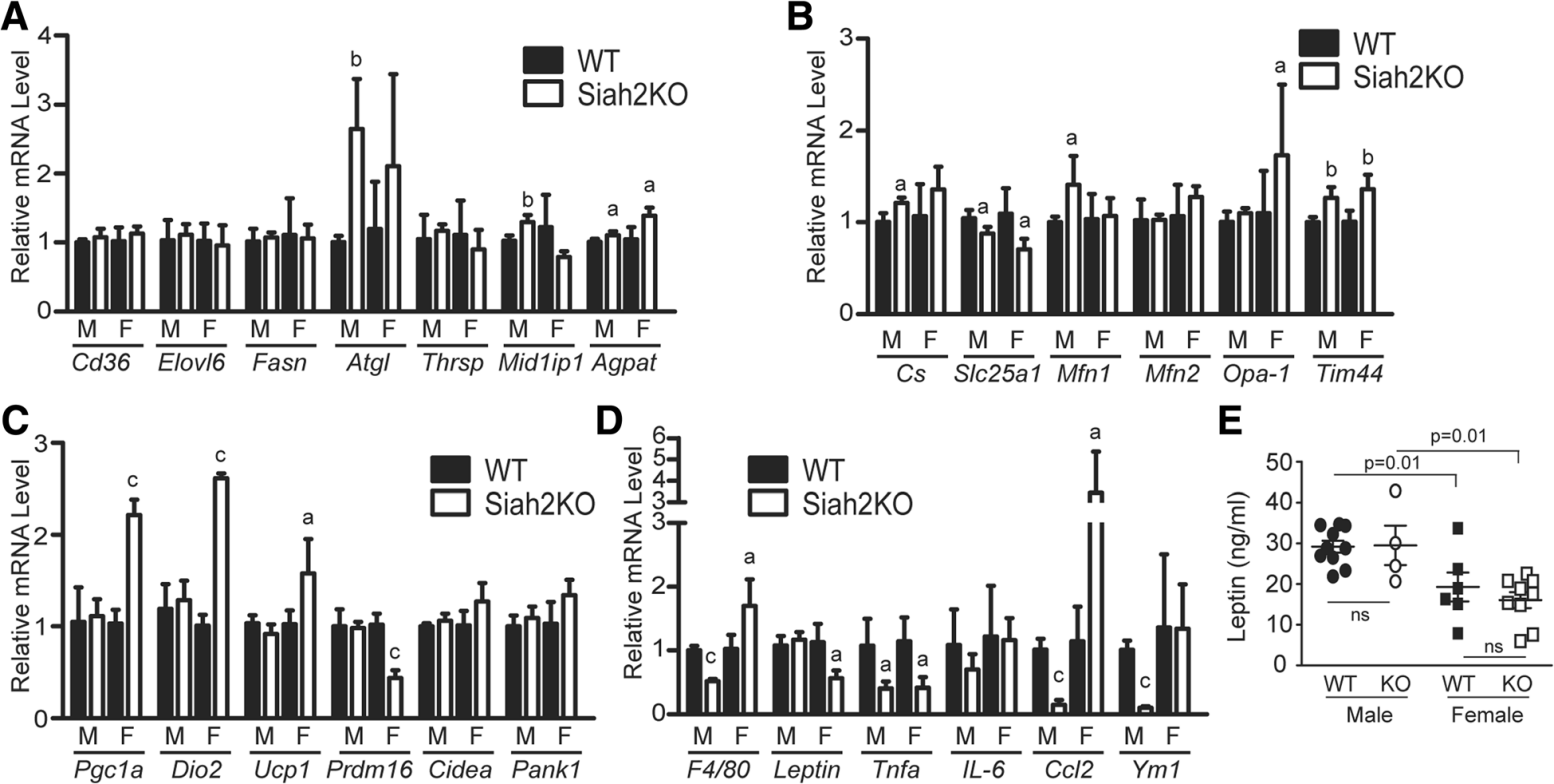
Siah2 regulates expression of markers of thermogenesis in female, but not male brown adipose tissue. Gene expression of markers of **a** lipid metabolism, **b** mitochondrial function, **c** thermogenesis, and **d** inflammation were assayed in brown adipose tissue of wild-type (WT) and Siah2KO (KO) male (M) and female(F) mice after 16 weeks on the HFD using real-time qRT-PCR. **e** Serum leptin levels were assayed in male and female wild-type (WT) and Siah2KO (KO) at 16 weeks on the HFD. Statistical significance was determined using a two-tailed, unpaired *t* test, a, *p* < 0.05, b, *p* < 0.01, c, *p* < 0.001; Siah2KO compared to wild-type within the same sex

In addition to microarray analysis, we investigated the expression of genes encoding proteins related to lipid metabolism, inflammation, and mitochondrial function. Of the lipid metabolism genes tested, adipose triglyceride lipase (*Atgl*) and Mid1ip1 (an acetyl-coenzyme A carboxylase-binding protein) transcripts were robustly upregulated in HFD-fed Siah2KO male BAT but not in females, whereas Agpat1 (acylglycerol-3-phosphate-O-acyltransferase-1) was upregulated in the HFD-fed Siah2KO animals independent of sex (Fig. 8a). Gene markers of mitochondrial function were regulated by Siah2 either in both sexes (*Slc25a1*, *Tim44*) or specifically in males (*Cs*, *Mfn1*) or females (*Opa-1*). In contrast, markers of thermogenesis displayed upregulation (*Pgc1a*, *Dio2*, *Ucp1*) or downregulation (*Pdrm16)* specifically in HFD-fed Siah2KO females (Fig. 8c). Markers of inflammation in brown fat displayed sex-by-genotype interactions (Fig. 8d). Thus, Siah2 deficiency corresponded to reduced mRNA expression of the macrophage-specific markers *F4/80* and *Ym1*, a M2-macrophage specific marker [41] in males, but increased expression in females. Among cytokine/chemokine genes, *Tnf alpha* expression was suppressed by Siah2 deficiency in both sexes, whereas *Ccl2* levels were significantly reduced Siah2KO males but highly upregulated in Siah2KO females. A similar sex-dependent effect was also observed for leptin, where loss of Siah2 led to reduced *Leptin* mRNA expression in females, but not in males. However, the brown fat specific reduction in *Leptin* transcripts did not correspond to reduced circulating levels of leptin protein in the HFD-fed Siah2KO females compared to wild-type, although leptin protein levels were significantly lower in females compared to males on the high-fat diet (Fig. 8e).

**Fig. 9:**
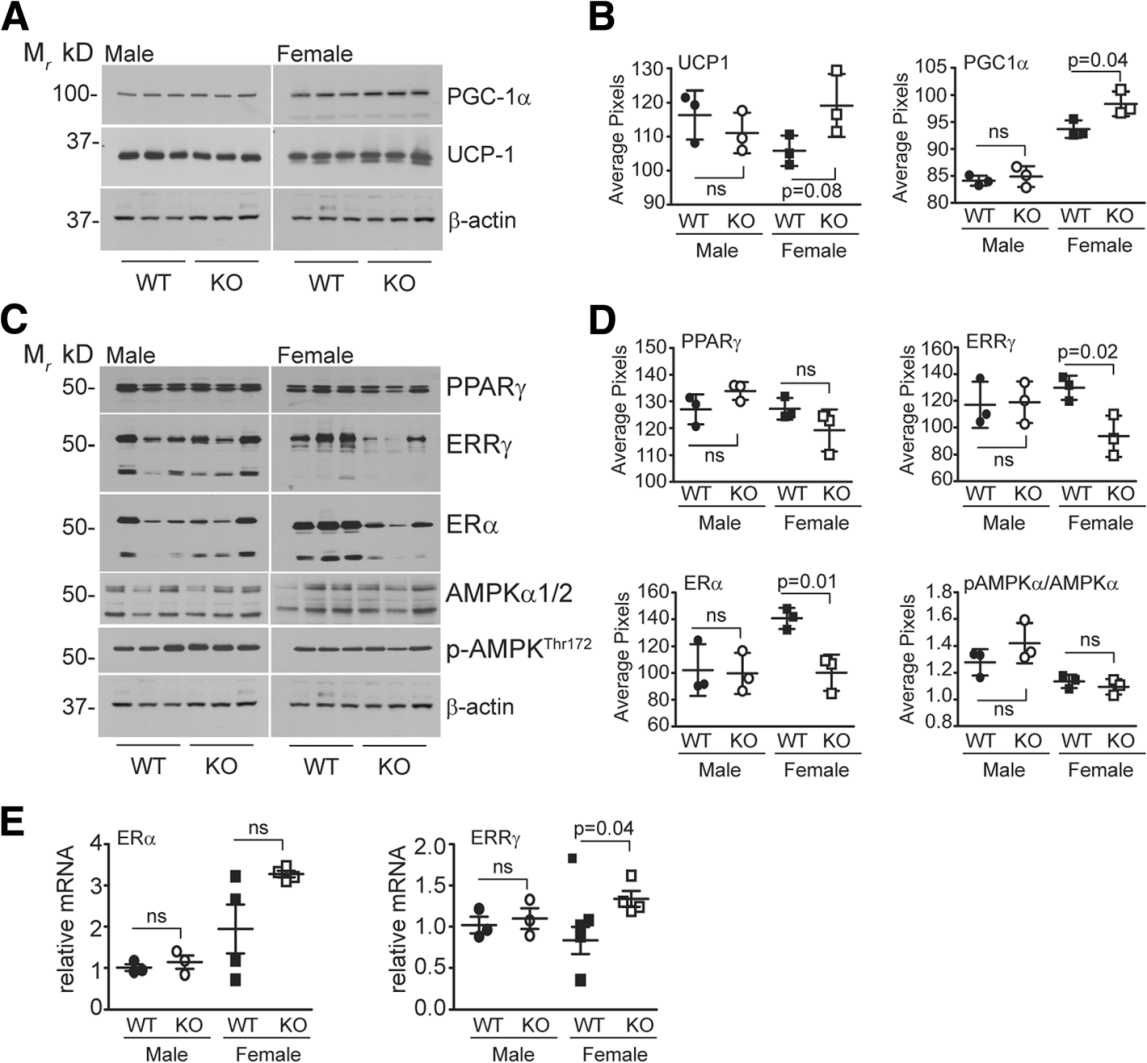
Loss of Siah2 upregulates thermogenic proteins, but downregulates ERα and ERRγ in brown adipose tissue independent of changes in gene expression in female, not male mice fed a HFD. **a** PGC1α and UCP1 and **c** PPARγ, ERRγ, ERα, AMPK1/2, and phosphorylated AMPK levels in brown adipose tissue were assayed via western blot analysis in male and female wild-type (WT) and Siah2KO (KO) mice after 16 weeks on the HFD and **b**, **d** quantified using Un-Scan-It software. **e** Gene expression of *Eralpha* and *Errgamma* was analyzed via real-time qRT-PCR. β-actin is included as a loading control in **a**, **c**. Statistical significance was determined using a two-tailed, unpaired *t* test

To determine if the Siah2-mediated changes in thermogenic gene expression corresponded to increased expression of the encoded proteins, we carried out western blot analysis of PGC-1α and UCP-1 expression in brown fat from the HFD-fed male and female mice. As shown in Fig. 9a, b, PGC1α and UCP1 were increased in HFD-fed females, but not in males. Thus, Siah2-mediated sex-dependent transcriptional regulation of thermogenic genes (Fig. 8c) correlates with the increased levels of PGC-1α and UCP1 protein in the brown adipose tissue of Siah2KO females. Given the evidence that Siah2 is an ERα transcriptional target [28, 42], we asked if ERα protein expression in brown fat is regulated by Siah2 deficiency. Notably, estrogens play a major role in regulating energy balance and thermogenesis through peripheral and central mechanisms [43], and ERα is also expressed in brown fat [44], although regulation of ERα signaling in brown fat per se is not well described. We anticipated ERα protein levels would be increased, corresponding to increased expression of ERα targets PGC1α and UCP1. However, ERα levels are substantially downregulated in the absence of Siah2 (Fig. 9c, d) in the HFD-fed females, but unchanged in males. We then assayed the expression of the estrogen-related receptor gamma (ERRγ), an orphan nuclear receptor closely related to the estrogen receptors that is highly expressed in brown fat and other oxidative tissue [45]. Recent studies show ERRγ is critical for maintaining the thermogenic capacity of brown fat [30] independent of PGC1α expression [46]. As with ERα, Siah2 deficiency educed ERRγ protein levels in female, but not male brown fat *r* (Fig. 9c, d). In contrast to protein levels, the transcript levels of *ERalpha* and *ERRgamma* gene expression appeared to increase in HFD-fed Siah2KO females, although the trend was either not significant or marginally significant (Fig. 9e).

Given the impact of estrogen-mediated hypothalamic AMPK activity on brown fat function, we assayed AMPK activity in the brown fat of the HFD-fed wild-type and Siah2KO mice. We found no Siah2-mediated effect on AMPK activity in the brown fat of the male or female mice (Fig. 9c, d). Finally, our earlier studies demonstrated that loss of Siah2 in obese male mice leads to increased PPARγ protein levels in gonadal fat [25] (Additional file 4 E). This prompted us to assay PPARγ levels with Siah2 depletion in brown fat. As shown in Fig. 9c, d, Siah2 does not regulate PPARγ in brown adipose tissue, although PPARγ trends down in the females and up in the males. While the limited number of samples assayed may be insufficient to capture significant increases in PPARγ in the brown fat of male mice, the results suggest Siah2-mediated regulation of select nuclear receptor protein levels depends on signaling events that are both fat depot-specific and sex-dependent. The results are summarized in Table 1.

**Table 1:** Summary of sex-related differences in Siah2KO adipose tissue

Siah2KO phenotype	Females (compared to WT and HFD unless otherwise noted)	Males (compared to WT and HFD unless otherwise noted)
Body composition		
Baseline fat mass (WT and KO)	Higher than males	Lower than females
Rate of gonadal fat mass gain (WT)	Lower than males	Higher than females
Rate of gonadal fat mass gain (KO)	Higher than males	Lower than females
Fat mass gain in BAT	Higher	Lower
BAT relative to body weight (WT and KO)	Higher than males	Lower than females
Adipocyte size	Larger	Larger
Percent fat mass at 4 months	Higher than males	Lower than females
Body weight, LFD	Higher	Higher
Body weight, HFD	Attenuated vs. WT	Higher
Biochemical measurements		
Glucose tolerance	Tolerant	Tolerant^23^
Insulin tolerance	Tolerant	Tolerant^23^
Fasting glucose	Unchanged	Lower^23^
Fasting insulin, LFD	Higher	Unchanged^23^
Fasting insulin, HFD	Unchanged	Lower^23^
Circulating free fatty acids	Unchanged (LFD), lower (HFD)	Lower^23^ (LFD and HFD)
Circulating triglycerides	Unchanged	Lower^23^
Inflammation and fibrosis markers in gWAT		
Crown-like structures	Fewer	Fewer
Adipose fibrosis	Lower	Lower
Macrophage marker (F4/80, CD68, CD11b)	No change	Lower^23^
M1-macrophage marker (CD11c)	Lower	Lower^23^
M2-macrophage marker (Ym1, Arg1, IL4)	Higher	Higher (Arg1)^23^
Pro-inflammatory markers (TNFα, IL6, Ccl2, Ccr2)	Unchanged	Lower^23^
Pai-1, Saa3 gene expression	Unchanged	Lower^23^
Gdf3 gene expression	Lower	Lower^23^
Thermogenesis markers in BAT		
ER-α/ERR-γ gene expression	Higher	No change
ER-α/ERR-γ protein expression	Lower	No change
PCG1α, Dio2, Ucp1 gene expression	Higher	No change
PCG1α, Dio2, Ucp1 protein expression	Higher	No change

*BAT* brown adipose tissue, *gWAT* gonadal white adipose tissue, *LFD* low-fat diet, *HFD* high-fat diet, *KO* Siah2 global knockout, *WT* wild-type

^23^Data reported in reference [23]

## Discussion

Earlier studies of the impact of Siah2 deficiency carried out in a male mouse model of diet-induced obesity showed the ubiquitin ligase Siah2 functions at the intersection of adipose tissue inflammation and insulin resistance in obesity. In the obese male mice, loss of Siah2 promotes lipid storage in hypertrophied adipocytes and reduces adipose tissue inflammation that leads to insulin resistance [25]. The HFD-fed male Siah2-deficient mice are a model of metabolically healthy obesity, a phenotype more typically associated with females [3] and attributed to the anti-inflammatory properties of estrogens [16]. The impact of Siah2 on adipose tissue inflammation coupled with estrogen-mediated regulation of Siah2 gene expression [28] prompted us to ask if there are sex-dependent effects of Siah2 deficiency on adipose tissue function in obesity. As found in the obese male mice, Siah2 in the high-fat-fed female mice regulates the relationship between white adipose tissue expansion via hypertrophy, adipose tissue inflammation, and insulin sensitivity. However, unlike the HFD-fed male mice [25], Siah2 deficiency in the HFD-fed females does not broadly dampen proinflammatory macrophage, cytokine, and chemokine expression. Instead, reduced crown-like structures in Siah2-deficient high-fat-fed females was associated with differential upregulation of markers of homeostatic resident macrophage and chemokines that promote alternative activation of macrophage. Genes encoding secreted factors such as TNFα and SAA3, that are generally associated with adipose tissue inflammation and insulin resistance [47], were also upregulated in the gonadal fat of HFD-fed Siah2KO female mice, despite their insulin sensitivity. This does not occur in obese Siah2KO male mice [25], but it agrees with recent evidence that SAA3 has anti-inflammatory properties and promotes a shift in macrophage toward a M2-like phenotype in adipose tissue [48]. Increased *Tnf alpha* mRNA in the context of a shift toward M2-like markers is also consistent with a positive role for pro-inflammatory stimulus in maintaining healthy adipose tissue as lipid storage capacity is challenged [49]. Thus, loss of Siah2 in female gonadal fat may differentially promote activation of resident M2-like macrophages and enhance adipose tissue remodeling to accommodate increased lipid storage demands in a sex-related manner.

In agreement with Wu et al. [50], we found that diet-induced fat expansion in the female gonadal fat occurred by increasing both hypertrophies of existing adipocytes and increasing the number of adipocytes while the male gonadal fat expanded by hypertrophy alone. In contrast, the inguinal and brown fat expanded solely by increasing the size of existing adipocytes in both sexes. Although the loss of Siah2 altered gene expression of inflammation markers, it did not change the mode of adipose tissue expansion in the HFD-fed females. In contrast, Siah2 deficiency significantly reduced the number of adipocytes in the white fat (and trending down in the BAT) of the HFD-fed males, lending further support for sex-related differences in the role of Siah2 in regulating the relationship between fat mass expansion and adipose tissue inflammation with obesity in white and brown fat.

Adipose tissue inflammation also occurs in obese brown adipose tissue as brown fat accumulates large unilocular lipid droplets characteristic of white adipose tissue in male mice [21]. Although leptin-mediated signaling has been implicated in brown fat inflammation when white fat-like unilocular lipid droplets accumulate in the brown fat [21], loss of Siah2 appears to disrupt this connection as “whitening” of the brown fat in the HFD-fed wild-type male mice (and to a less extent in the Siah2KO male mice), and elevated leptin mRNA and protein levels were not accompanied by increased markers of inflammation. In contrast, reduced leptin mRNA and protein levels in the HFD-fed females were associated with robustly increased mRNA expression of a pro-inflammatory chemokine marker (*Ccl2*) in the Siah2KO females.

While a relatively low number of genes are differentially regulated between the sexes in brown fat by Siah2 with a HFD challenge, decreased expression of genes supporting fatty acid oxidation in the Siah2KO males and lower levels of genes controlling lipogenesis in Siah2KO females are consistent with the morphological data with Siah2 deficiency in both sexes. Lipolysis (*Atgl*) is likely increased by Siah2 deficiency with a short fast, but other markers of lipid metabolism are not substantially regulated by sex or genotype. However, we cannot rule out significant changes in lipid metabolism with Siah2 deficiency in the brown fat of either sex given the extensive post-translational regulation of lipid metabolism exemplified by ATGL-mediated control of lipid storage and release [51].

Most striking are the sex- and genotype-dependent differences related to inflammation and thermogenesis. The effect of Siah2 deficiency on brown fat inflammation in the HFD-fed males indicates generally reduced inflammatory responses to increased brown fat whitening in the obese Siah2KO males, as we observed in the male Siah2KO white adipose tissue. However, the pattern is more nuanced in females where the brown fat morphology is devoid of significant whitening or crown-like structures although proinflammatory markers are transcriptionally upregulated with Siah2 deficiency in the HFD-fed females. The Siah2-dependent increase in mRNA expression of the macrophage and proinflammatory chemokine markers in a setting of morphologically healthy brown fat in the females is consistent with a role for Siah2 in estrogen-mediated accelerated resolution of high-fat-induced inflammatory processes.

Minimal whitening of female brown fat was coupled with robust upregulation of thermogenic genes with a corresponding increase in PGC1α and UCP1 protein levels in the Siah2-deficient HFD-fed females. This effect is absent in the male brown fat, indicating Siah2 in brown fat suppresses diet-induced thermogenic responses in a sex-dependent manner. Estrogens are well-described as acting centrally via estrogen receptor alpha (ERα)-mediated inhibition of hypothalamic AMPK activity to stimulate brown fat thermogenesis via upregulation of *Ucp1* and *Pgc1a* mRNA [29]. While the Siah2 deficiency model is a global deletion of Siah2, expression of Siah2 is not detected in the hypothalamus of wild-type C57BL/6 mice although it is found in the olfactory bulb and cerebellum [52]. This suggests the effect of Siah2 on thermogenic markers more likely occurs peripherally. ERα is also expressed in male and female brown fat, but there is less data on the direct effects of estrogens on brown fat function.

The sex-related effect of Siah2 on thermogenic gene expression in the female brown fat did not depend on AMPK signaling in brown fat, and unexpectedly, loss of Siah2 in the HFD-fed female brown fat substantially reduced expression of ERα and ERRγ proteins while reductions in PPARγ protein levels were not statistically significant. Moreover, reduced PPARγ protein expression contrasts with the effect of Siah2 deficiency on PPARγ protein levels in white fat of HFD-fed obese male mice [25] and (Additional file 4 E), suggesting both sex- and fat depot-specific effects of Siah2 in nuclear receptor protein levels. Nonetheless, Siah2 deficiency stimulates expression of thermogenic genes while decreasing the levels of the transcription factors that regulate thermogenic gene expression. This most likely occurs via a post-transcriptional mechanism as *ERα* and *ERRg* gene expression increased with loss of Siah2, consistent with a feedback loop to maintain protein expression in the context of accelerated receptor turnover [53].

In a series of studies, the O’Malley group established that ligand-dependent activation of ERα is coupled to proteasome-dependent degradation of the activated receptor [54, 55]. Our data demonstrates the enhanced degradation of ERα and ERRγ in the absence of Siah2. This is unexpected given the existing paradigm for the role of Siah2 as part of a nuclear receptor corepressor complex that restrains the activity of nuclear receptors. According to this scenario, Siah2 interacts with the corepressor N-CoR and promotes ligand-dependent nuclear receptor activity by targeting N-CoR for proteasomal degradation [56]. If Siah2 were regulating ER*α* and ERRγ protein levels by dismissing a corepressor, loss of Siah2 is expected to increase ERα and ERRγ protein levels as N-CoR remains bound to the receptor, disrupting ligand-dependent activation and proteasome-dependent degradation of the receptors as well as N-CoR. Our result suggests Siah2 acts by a different mechanism to restrain activation and turnover of ERα and ERRγ to control brown fat thermogenesis in females.

However, our study has several limitations. While the data supports a sex-related role for Siah2 in adaptive thermogenesis, we did not directly assay thermogenesis or energy expenditure and the ability to adapt to cold temperatures in the wild-type and Siah2-deficient male and female mice. We also did not carry out the studies of Siah2 deficiency in the absence of either ERα or ERRγ to provide conclusive evidence of a role for Siah2 in estrogen-mediated regulation of brown fat function. As we show in this study, transcriptional changes may not reflect (or may be inversely related to) post-transcriptional levels of the encoded protein. Thus, transcriptional changes in inflammatory markers or lipid metabolism genes do not provide direct evidence of changes in the levels or activity of the encoded protein. We attempt to overcome this limitation by assaying the mRNA expression of a range of inflammatory markers coupled with morphological changes and selected protein expression to gain an understanding of a regulatory pattern indicative of adipose tissue inflammation. Finally, although our study is carried out in mice, the relatively high levels of brown fat observed in the female mice mirrors the higher levels found in women compared to men. The female mice also show resilience to the metabolic effects of adiposity that occur in premenopausal women. These similarities suggest the mouse model of Siah2 deficiency may provide important and relevant mechanistic insights into sex-related differences in men and women in response to obesity.

## Conclusion

The ubiquitin ligase Siah2 is an important mediator of the relationship between adipose tissue expansion via hypertrophy, adipose tissue inflammation, and impaired glucose tolerance in male and female mice that are chronically over-fed with a high-fat diet. There are similarities between the sexes in the impact of Siah2 deficiency on morphological evidence of white adipose tissue inflammation. However, important sex-related differences in expression of genes encoding markers of inflammation suggest the underlying mechanism responsible for reduced inflammation in the adipose tissue differs between HFD male and females. The effect of Siah2 deficiency on adipose tissue function extends to brown fat with substantial sex-related effects of Siah2 on the regulation of thermogenic markers in the brown fat of the HFD-fed mice. The stimulation of thermogenic gene and protein expression and regulation of ERα and ERRγ protein levels only in the Siah2-deficient females suggests that Siah2 restrains the impact of ERα and ERRγ proteins on brown fat function in females, but not in male mice. This finding further underscores the sex-dependent roles of Siah2 in key metabolic tissues when challenged with chronic excess calorie intake.

## Additional files

Additional file 1: This is an excel file (.xlsx) titled Supporting Information Related to Gene Expression Analysis (XLSX 12 kb)

Additional file 2: This is an excel file (.xlsx) titled Antibody Information (XLSX 11 kb)

Additional file 3: This is a tiff file titled Additional Information on White and Brown Fat Morphology (TIF 6267 kb)

Additional file 4: This is a tiff file title Additional Information on Metabolic and Inflammatory markers and PPARγ protein expression is Siah2-deficient male mice (TIF 233 kb)
