# Correction to: Siah2 modulates sex-dependent metabolic and inflammatory responses in adipose tissue to a high-fat diet challenge

**DOI:** 10.1186/s13293-019-0252-8

**Published:** 2019-07-22

**Authors:** Sujoy Ghosh, Jessica L. Taylor, Tamra M. Mendoza, Thanh Dang, David H. Burk, Yongmei Yu, Gail Kilroy, Z. Elizabeth Floyd

**Affiliations:** 1 Pennington Biomedical Research Center, Baton Rouge, LA 70808 USA; 2 Cardiovascular and Metabolic Disease Program and Center for Computational Biology, Duke-NUS Graduate Medical School, Singapore, Singapore

**Correction to: Biol Sex Differ**

**https://doi.org/10.1186/s13293-019-0233-y**

Following publication of the original article [1], the authors reported that Additional file 1 was incorrect. The corrected Additional file 1 is given below.

## Additional file

Additional file 1 Related to Gene Expression Analysis. (XLSX 13 kb)
