# Supplementary figures and images for: Siah2 modulates sex-dependent metabolic and inflammatory responses in adipose tissue to a high-fat diet challenge
Source: Biol Sex Differ. 2019 Apr 15;10:19. doi: 10.1186/s13293-019-0233-y (PMC6466809; doi:10.1186/s13293-019-0233-y)

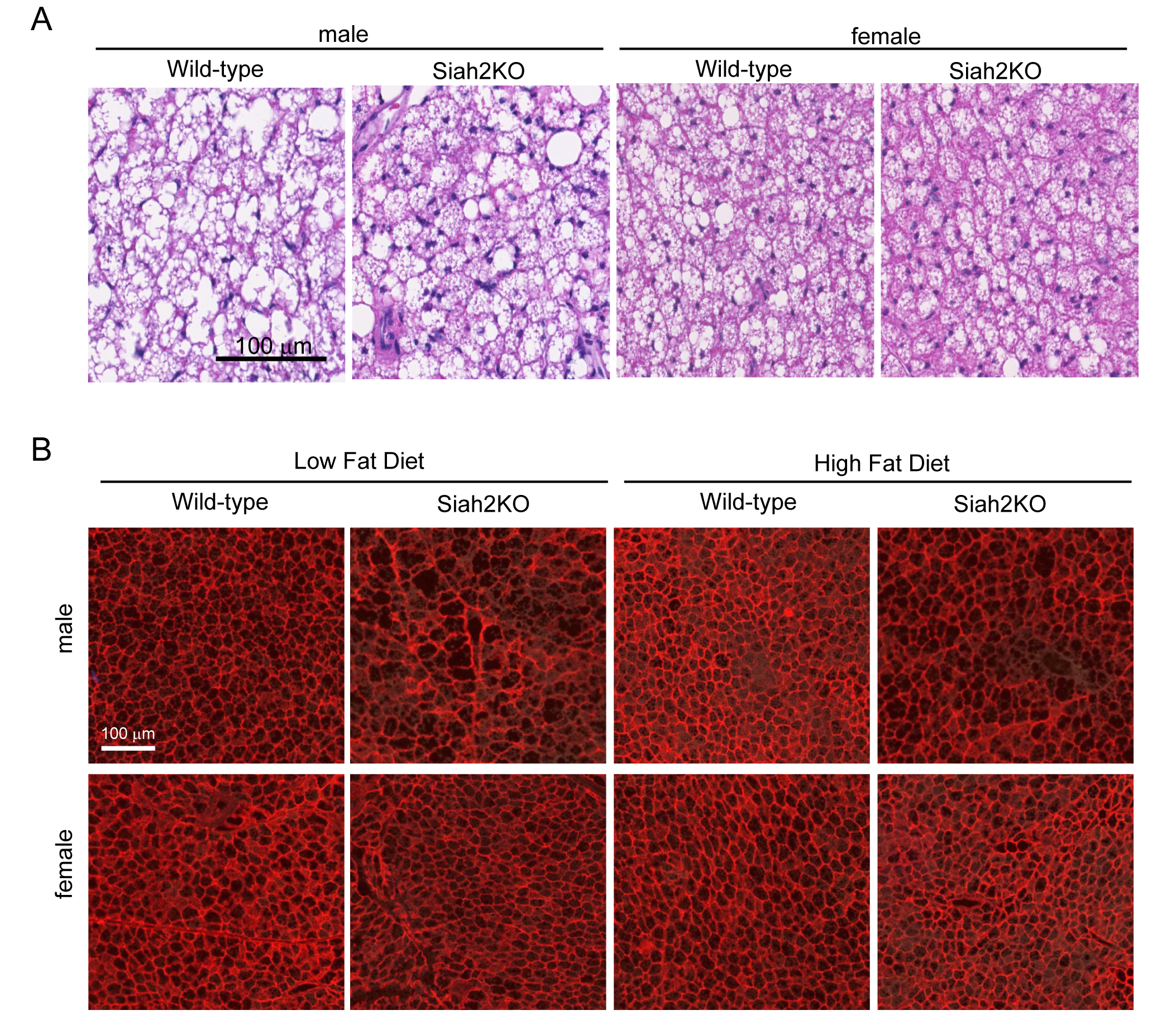

Supplement: Supplementary file 3 — This is a tiff file titled Additional Information on White and Brown Fat Morphology (TIF 6267 kb) [file 13293_2019_233_MOESM3_ESM.tif]

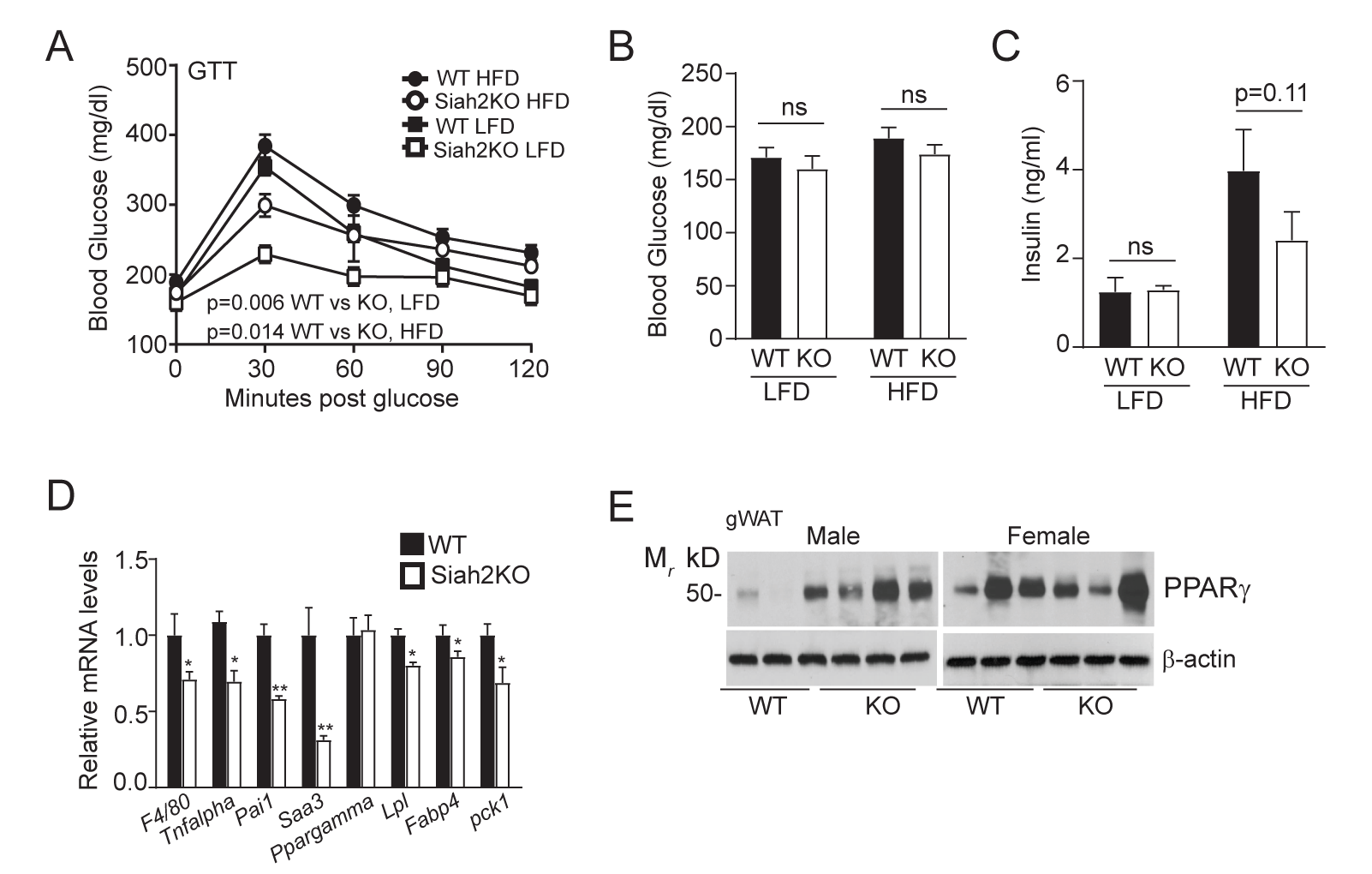

Supplement: Supplementary file 4 — This is a tiff file title Additional Information on Metabolic and Inflammatory markers and PPARγ protein expression is Siah2-deficient male mice (TIF 233 kb) [file 13293_2019_233_MOESM4_ESM.tif]
